# Transcriptomic and Phenotypic Responses of Cucumber Trichome Density to Silver Nitrate and Sodium Thiosulfate Application

**DOI:** 10.3390/ijms26031298

**Published:** 2025-02-03

**Authors:** Muhammad Ahmad, Sen Li, Li Shan, Songlin Yang, Yaru Wang, Shanshan Fan, Menghang An, Yingqi Shi, Yifan Xu, Tiantian Pei, Xinyue Ma, Yibing Zhao, Hao Xue, Xingwang Liu, Huazhong Ren

**Affiliations:** 1 Department of Vegetable Science, College of Horticulture, China Agricultural University, Beijing 100193, China; ahmad31710@gmail.com (M.A.); bs20233171035@cau.edu.cn (S.L.); yangsonglin@cau.edu.cn (S.Y.); s20223172964@cau.edu.cn (S.F.); anmenghang@cau.edu.cn (M.A.); shiyingqi0426@cau.edu.cn (Y.S.); sy20243173343@cau.edu.cn (Y.X.); pei8936@cau.edu.cn (T.P.); maxiny@cau.edu.cn (X.M.); sy20243173443@cau.edu.cn (Y.Z.); xuehao@cau.edu.cn (H.X.); 2 Sanya Institute of China Agricultural University, Sanya 572000, China; shanli01@cau.edu.cn (L.S.); b20213170888@cau.edu.cn (Y.W.)

**Keywords:** *Cucumis sativus*, regulation, morphology, sodium thiosulphate, silver nitrate, RNA-seq analysis, foliar application

## Abstract

Cucumber (*Cucumis sativus* L.) is one of the most widely cultivated crops worldwide and is valued for its nutritional, economic, and ecological benefits. The regulation of defense mechanisms against herbivores, along with osmotic loss and environmental regulation, is greatly affected by trichomes in cucumbers. In this study, we attempted to characterize trichomes and examined fruit physiological and transcriptome profiles by RNA sequencing in cucumber breeding lines 6101-4 and 5634-1 at three stages of fruit development through foliar application with a combination of silver nitrate (AgNO_3_) and sodium thiosulfate (Na_2_S_2_O_3_) in comparison to non-treated controls. Notable increases in the number of trichomes and altered forms were observed for both inbred cultivars 6101-4 and 5634-1 against foliar application of chemical substances. RNA-seq analysis was performed to identify differentially expressed genes (DEGs) involved in multiple pathways in cucumber trichome formation. The enrichment of differentially expressed transcripts showed that foliar application upregulated the expression of many stress-responsive and trichome-associated genes including plant hormone signal transduction, sesquiterpenoid and triterpenoid biosynthesis, and the mitogen-activated protein kinase (MAPK) signaling pathway. The dominant regulatory genes, such as *allene oxide synthase* (*AOS*) and *MYB1R1* transcription factor, exhibited significant modulations in their expression in response to chemical application. The RNA-seq results were further confirmed by RT-PCR-based analysis, which revealed that after chemical application, the dominant regulatory genes, such as *allene oxide synthase* (*AOS*), *PTB 19*, *MYB1R1*, *bHLH62-like*, *MADS-box* transcription factor, and *salicylic acid-binding protein 2-like*, were differentially expressed, implying that these DEGs involved in multiple pathways are involved the positive regulation of the initiation and development of trichomes in *C. sativus*. A comparison of trichome biology and associated gene expression regulation in other plant species has shown that silver nitrate (AgNO_3_) and sodium thiosulfate (Na_2_S_2_O_3_) are also responsible for hormonal and signaling pathway regulation. This study improves our knowledge of the molecular mechanisms involved in *C. sativus* trichome development. It also emphasizes the possibility of utilizing chemical composition to modulate *C. sativus* trichome-related characteristics of *C. sativus*, leading to the improvement of plant defense mechanisms as well as environmental adaptation.

## 1. Introduction

*Cucumis sativus* L. is a member of the Cucurbitaceae family, which includes many economically important crops called cucurbits. These include melon (*Cucumis melo* L.), watermelon (*Citrullus lanatus* L.), squash and pumpkin (*Cucurbita* spp.), bitter gourd (*Momordica charantia* L.), and bottle gourd (*Lagenaria siceraria* L.) [1,2]. There was 185.41 billion kilograms (BKg) of worldwide cucumber production in 2024, of which China led at 77.3 BKg (FAO, 2024). Cucumbers contain many important nutrients, including iron, calcium, proteins, minerals, and vitamins [3]. Cucumber fruits have antioxidant properties because they contain flavonoids, polyols, and polysaccharides that delay aging, help immunity, and improve mental health. Cucumbers contain vitamins B and C, which can prevent liver infections and hinder the growth of abnormal and undifferentiated cells responsible for tumors and brain injuries [4].

Cucumber (*Cucumis sativus* L.) is most significant vegetable crop; the stems and leaves, flowers, branches, fruits, and tendrils are covered with trichomes. In early fruit maturation, deep longitudinal ridges spiraling over the fruited surface are observed, while the fruit trichomes are inconspicuously numerously and randomly positioned with reference to these ridges [5,6]. These spines, or fruit trichomes, of cucumbers are complex formations which represent the outgrowth of epidermic cells. As is expected from many other plant species, cucumber fruit trichomes are morphologically heterogeneous in terms of the shapes, sizes, structural organization, and ability to secrete [6,7,8]. Fruit spines are also fruit quality traits that affect the fruit value or commercial worth of the cucumber fruit [8]. Trichomes could also have significant roles in the defense of plants against biotic and/or abiotic stresses, including heat, low-temperature stress, or high UV radiation, and insect feeding [9,10]. Non-glandular hooked trichomes are considered specific structures that are efficient at trapping a number of herbivores as well as their antagonists [11,12]. Glandular trichomes (GTs) are special structures that secrete a variety of compounds including essential oils, resins, and other secondary metabolites. They possess antibacterial properties, attract pollinators, and prevent herbivores [13]. They are known for their role in the synthesis of useful phytochemicals, such as in tomatoes and cucumbers, which have important economic and medicinal value [14].

The sex characteristics of cucumber are related to genetic, chemical, and environmental conditions [15]. Plant foliage can be treated with silver nitrate and gibberellin, which contribute to the induction of male flowers in plants, as they can affect gene expression when applied to the leaves of the plant [16,17]. These compounds can disrupt plant hormone levels and increase the expression of genes for flowering and sex determination. In 1976, it was found that silver ions can block ethylene production and force open the male sperm channel (female flowers require this hormone to grow) [18,19]. AgNO_3_ has been used extensively for both agronomic and research purposes. Ethylene receptors are thought to occupy the copper binding site and interact with ethylene, but Ag+ inhibits the ethylene response [20,21,22]. Additionally, ethylene controls cucumber floral sex determination [23,24] by interfering with another developmental process and inducing femaleness upon exogenous application or increased endogenous ethylene levels. Endogenous levels of growth hormones determine sex expression in cucumber, but sex can be triggered by the exogenous application of growth regulators [25]. Gibberellins, for instance, stimulate genes related to male floral development by promoting male floral organ growth and inhibiting certain genes involved in floral development. The treatment of monoecious and gynoecious cucumber plants with ethephon and silver thiosulfate has also been shown to induce female and male flower development, respectively [26].

Although various studies have been carried out to determine the effect of silver nitrate (to analyze the flower quantity of cucumbers [27]) and silver thiosulfate on male flowering induction in cucumber [26], their effect on the genetic regulation of trichome regulation in cucumber remains unexplored. In this study, we used these two chemicals in combination as foliar applications to check their effects on fruit trichome density at three different stages: 5 days before anthesis, the day of anthesis, and 5 days after anthesis. The reason behind the choice of these stages is that a significant number of genes related to trichome formation and differentiation are highly active, making them ideal for studying how various treatments may influence trichome density and morphology. The application of these chemicals induces sex expression, increases the density of cucumber fruits, and affects both morphological and molecular levels. This study aimed to investigate the effects of male-flower-inducer chemicals on trichome initiation and development on cucumber fruits by sequencing both inbred lines and analyzing the sequencing data. Taken together, our results provide a basis for further understanding the mechanisms of trichome development and the relationship between trichomes and foliar application of chemicals.

## 2. Results

### 2.1. Trichome Density and Morphological Variations in Cucumber Genotypes at 5 DBF Stage

**Figure 1:**
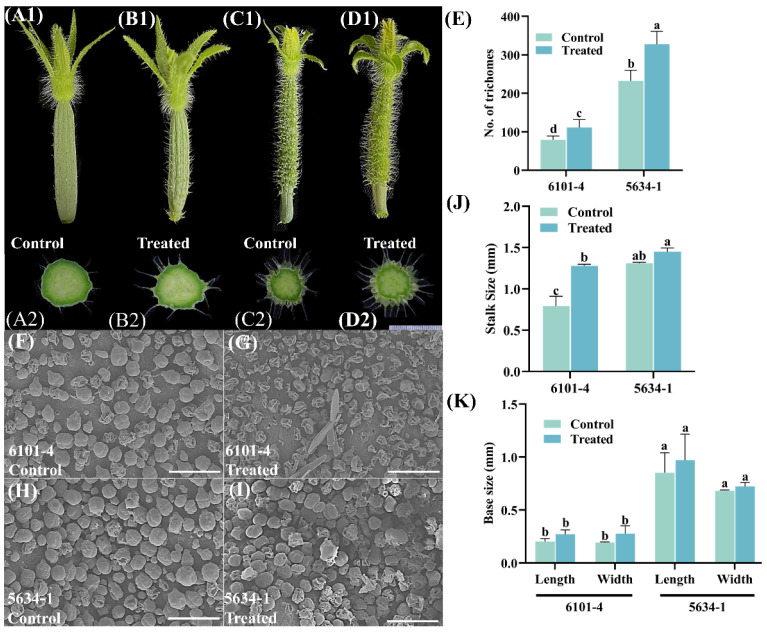
Morphological and microscopic analysis of trichomes in control and treated plants of lines 6101-4 and 5634-1 at 5 DBF. (**A1**–**D1**) Morphological comparison of trichomes from control and treated plants in lines 6101-4 (**A1**,**B1**) and 5634-1 (**C1**,**D1**). Cross-sectional views of immature fruits (**A2**–**D2**) of the trichomes illustrate the differences in density and length between treated and control plants. (**F**–**I**) Scanning electron microscopy (SEM) and scale bar represent 200 µm. The bar charts on the right side represent quantitative analyses of the trichome density (**E**) and trichome stalk size (**J**) for both lines under control and treated conditions. (**K**) Base measurements (error bars represent values of *n* = 8 samples and different letters represent significant differences at *p* ≤ 0.05).

Application of foliar treatment had significant effects on trichome characteristics in cucumber lines 6101-4 and 5634-1 at the 5 DBF stage with significant differences in trichome density, stalk size, and base measurements. Different samples were compared in terms of trichome density (Figure 1A1–D1). In line 6101-4 (Figure 1A1,B1), treated plants showed a significant increase in trichome density with a trichome number of approximately 100 in control plants up to approximately 150 in treated plants (*p* < 0.05). A 50% increase in trichome number was a clear indication of the response to foliar treatment to enhance trichome density (Figure 1E). In line 5634-1 (Figure 1C1,D1), the number of trichomes increased significantly from approximately 250 in the control plants to 350 in the treated plants (*p* < 0.0001), with a 40% increase (Figure 1E). Taken together, these results indicate that foliar application of trichomes consistently increases trichome density across diverse genetic backgrounds, providing an efficient means for increasing trichome-related traits.

In terms of trichome stalk size, the response to foliar application differed between the two lines. Stalk size increased significantly (*p* < 0.0001) from 1.0 mm in the control plants to 1.5 mm in the treated plants in the 6101-4 line. The 50% increase in stalk size suggested that the treatment not only increased the number of trichomes but also increased trichome stalk size, potentially improving trichome function (Figure 1J). In contrast, there was no significant difference in stalk size between control and treated plants in the 5634-1 line (Figure 1J), where the stalk size in control and treated plants was about 1.5 mm (mean). This suggests that although foliar application increases trichome density, its effect on stalk size is genotype-specific, emphasizing the role of genetic background in determining phenotypic responses. Quantitative observations are supported by photographic evidence and relevant SEM results, which visually show increased trichome density in treated plants vs. control plants (Figure 1) in both lines. The statistical data were corroborated by these images, which clearly demonstrated the phenotypic differences induced by foliar application. Trichomes were also visibly increased in treated plants in line 6101-4, and both density and stalk size were enhanced. Additionally, measurements on that change were made as further base measurements were taken, including the base length and base width; however, these were not significant (Figure 1K). Foliar application significantly increased trichome density in cucumber lines 6101-4 and 5634-1. It also increased the trichome stalk size in 6101-4 and had no effect on stalk size in 5634-1. Treatment did not affect the base measurements of trichomes in either line. These results reveal the differential phenotypic response of cucumber lines to foliar applications and provide important information on targeted agronomic practices that can increase desirable traits in cucumber cultivars.

### 2.2. Trichome Density and Morphology of Immature Fruits at Flowering (DF)

**Figure 2:**
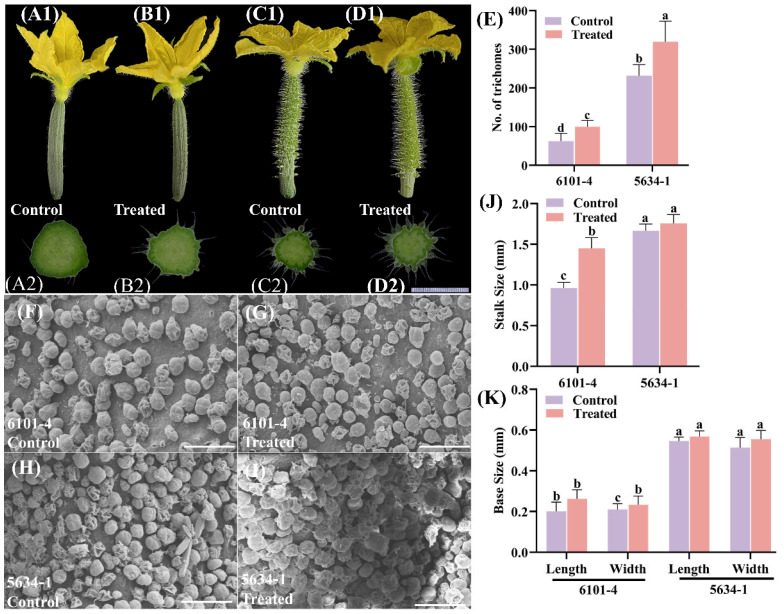
Comparison of floral morphology, trichome density, and stalk size between control and treated plants in lines 6101-4 and 5634-1 at 0 DBF. (**A1**–**D1**) Morphological comparison of fruit trichomes from control and treated plants in lines 6101-4 (**A1**,**B1**) and 5634-1 (**C1**,**D1**). Immature fruit cross-sectional views (**A2**–**D2**) of the trichomes highlight differences in length and density between treated and control plants. (**E**) Quantitative analysis of trichome density. (**F**–**I**) SEM images of the trichome and scale bar represent 200µm. (**J**) Stalk size comparison between control and treated plants in both lines. (**K**) Base measurements. Error bars represent values of *n* = 8 samples and different letters represent significant differences at *p* ≤ 0.05.

Foliar treatment significantly affected the trichome phenotype in cucumber lines 6101-4 and 5634-1 at 0 DBF. In the 6101-4 line, treated plants showed a considerable increase in trichome density. This can be seen in Figure 2A1–D1. The number of trichomes increased significantly from approximately 100 in the control plants to 150 in the treated plants (*p* < 0.05). This 50% increase underscores the effectiveness of foliar application in enhancing trichome production (Figure 2A1,B1,E). For the 5634-1 line, the number of trichomes also increased significantly, from approximately 250 in the control plants to 350 in the treated plants (*p* < 0.0001), representing a 40% increase (Figure 2C1,D1,E). These findings indicate that foliar application enhances trichome density at other stages of development, proving that it can be used to study novel traits related to trichome development and initiation (Figure 2). The effect of the foliar application on trichome stalk size differed between the two lines (Figure 2J). In the 6101-4 line, there was a significant increase in stalk size from 1.0 mm in control plants to 1.5 mm in treated plants (*p* < 0.0001). This 50% increase in stalk size suggests that the treatment not only boosts trichome numbers but also enhances their structural size, potentially improving their functional capacity (Figure 2J). Conversely, in the 5634-1 line, no significant difference in stalk size was observed between control and treated plants, both maintaining a mean stalk size of approximately 1.5 mm (Figure 2J). This indicates that, while foliar application increases trichome density, its effect on stalk size is genotype-specific, underscoring the importance of genetic background in modulating phenotypic responses. The bases of trichomes were also determined. This includes the base length and width, similar to the previous stage. These results were not statistically significant (Figure 2K).

Photographic evidence supports these quantitative findings, showing an increased density of trichomes in treated plants compared to control plants (Figure 1 and Figure 2) for both 6101-4 and 5634-1. The statistical data corroborated the phenotypic differences illustrated in the images (Figure 2E,J). The treated plants displayed visibly greater trichome numbers, especially in line 6101-4, where trichome density and stalk size increased. The differential responses between the two cucumber lines indicate a complex plant response to exogenous treatments, and the genetic background determines this response. Finally, foliar application significantly increased trichome density in cucumber lines 6101-4 and 5634-1. In addition, it increased the size of trichome stalks in the 6101-4 line (Figure 2J) but had no effect on stalk size in the 5634-1 line. The treatment had no effect on the base measurements of trichomes in either line (Figure 2K). These results highlight the differential phenotypic responses of cucumber lines when exposed to foliar applications and should be informative for the design of targeted agronomic practices to improve desirable traits in selected cucumber cultivars.

### 2.3. Trichome Density and Morphology of Fruits 5 Days After Flowering (5 DAF)

**Figure 3:**
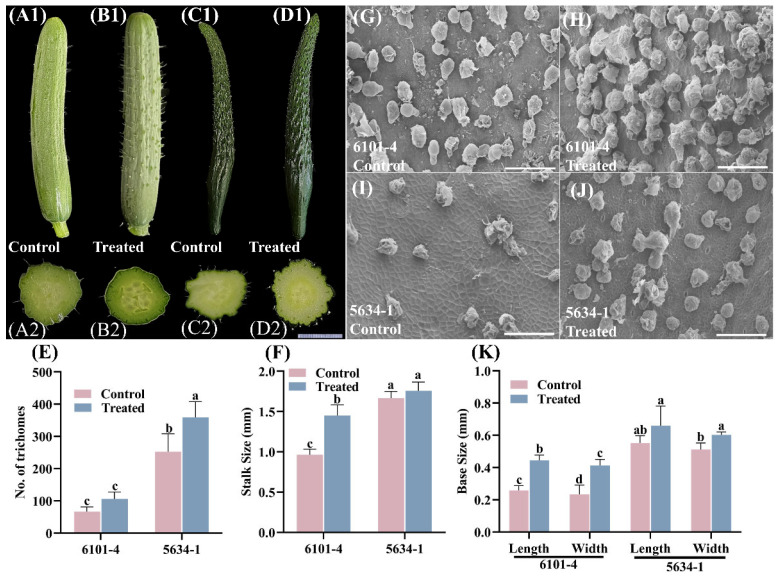
Morphological and SEM analysis of cucumber fruits from control and treated plants of lines 6101-4 and 5634-1 at 5 DAF. (**A1**–**D1**) Morphological comparison of cucumber fruits from treated and control plants in lines 6101-4 (**A1**,**B1**) and 5634-1 (**C1**,**D1**). Cross-sectional (**A2**–**D2**) views show the differences in trichome between treated and control plants. (**G**–**J**) SEM images of the epidermal surface of cucumber and scale bar represent 200 µm (**E**) highlighting trichome density (**F**) Stalk size comparison between control and treated plants in both lines. (**K**) Base measurements. Error bars represent values of *n* = 8 samples and different letters represent significant differences at *p* ≤ 0.05.

At the 5 DAF (5 days after flower open) stage, foliar treatment had varying impacts on trichome characteristics in cucumber lines 6101-4 and 5634-1. The treatments resulted in differential responses in trichome density, stalk size, and base measurements between the two lines. In the 6101-4 line, the number of trichomes did not show a significant increase in treated plants compared to control plants (Figure 3A1,B1,E). This suggests that at this stage, foliar application did not markedly influence trichome density in this line. Conversely, in the 5634-1 line, there was a significant increase in trichome density, with treated plants showing an increase from approximately 250 trichomes in control plants to 400 trichomes in treated plants (*p* < 0.0001) (Figure 3C1,D1,E). This substantial enhancement indicated a strong genotype-specific response to foliar application of increasing trichome density in the 5634-1 line (Figure 3E).

The stalk sizes of the trichomes also displayed differential responses. In the 6101-4 line, treated plants exhibited a significant increase in stalk size from approximately 1.2 mm in control plants to 1.8 mm in treated plants (*p* < 0.001) (Figure 3F). This increase underscores the ability of foliar treatments to enhance the structural attributes of trichomes in this line at that particular stage. However, in the 5634-1 line, no significant difference in stalk size was observed between treated and control plants, both maintaining a mean stalk size of approximately 1.8 mm (Figure 3F). This finding suggests that the effect of the treatment on trichome stalk size is not uniform across different genetic backgrounds. Finally, foliar application at 5 DAF strongly increased trichome density in the 5634-1 line but not in the 6101-4 line (Figure 3). In addition, trichome stalk size and base measurements increased in the 6101-4 line (Figure 3F,K), but not in the 5634-1 line. These results demonstrate genotype-specific responses of cucumber lines to foliar applications, which provides important information on fine-tuning agronomic practices for enhanced desirable traits in specific cucumber cultivars.

These quantitative findings are supported by photographic and SEM results, such that treated and control plants in both lines were visually demonstrated to have different trichome characteristics (Figure 3). Clearly, increased density and altered structural dimensions in treated plants are readily apparent in the 6101-4 line, where both density and base dimensions increased (Figure 3E,K). The observation of differential responses of the two cucumber lines at the 5 DAF stage indicates the complexity of plant responses to exogenous treatments and the key role of genetic background in these phenotypic changes. Genotype-specific effects of foliar treatment, potentially related to underlying genetic or physiological mechanisms, appeared to be at play in the 6101-4 line, reflected in significant increases in trichome density and structural dimensions. Furthermore, the response of the 5634-1 line, with an increase in trichome density without changes in stalk size or base measurements, implies different genetic or regulatory pathways.

### 2.4. Trichome Scanning Electron Microscopy (SEM) Analysis

The comparative analysis of trichome morphology between control and treated cucumber plants was conducted in both breeding lines, 6101-4 and 5634-1, using scanning electron microscopy (SEM). The SEM images further elucidate these differences, offering a higher-resolution view of the trichome surface (Figure 1F–I, Figure 2F–I and Figure 3G–J) in both 6101-4 and 5634-1 lines. The micrographs were captured at a range of resolutions from 1.00 mm to 500 µm. The findings indicated that cucumber fruits of both lines, 6101-4 and 5634-1, differed slightly from the control in appearance and texture, and noticeable differences were observed only at various stages of the plant, including morphology and trichome density in terms of glandular trichomes. Further, in the treated plants of both lines, glandular trichomes displayed enhanced structural features, including larger secretory heads, indicative of an increased secretion capacity. Glandular trichomes in the treated samples also showed changes in their surface texture and density, suggesting a modification in their protective function (Figure 1F–I, Figure 2F–I and Figure 3G–J). In both cases, the higher density of trichomes in lines 6101-4 and 5634-1 relative to the control suggests a major change in the processes of trichome initiation and development when grown under identical conditions. SEM images confirmed that this impact was at the cellular level, with dramatic differences in the surface morphology between the treated and control samples. This implies that the surface structure of the fruits of cucumber may be changed by the treatment or may be changed early in its growth or development. Collectively, these results indicate that the treatment has a significant effect on the development and morphology of both glandular and non-glandular trichomes in cucumber plants. The observed alterations in trichome structure and density suggest that the treatment may influence the plant’s defensive mechanisms and possibly enhance the secretion of bioactive compounds.

### 2.5. Overview of RNA Sequencing

To investigate responses in the fruit peel of cucumber fruit following foliar application of silver nitrate and sodium thiosulfate, cultivars of *C. sativus* (6101-4 and 5634-1) were used as experimental materials. For RNA sequencing, only healthy cucumber plants with similar vigor were chosen and had not shown any symptoms of disease or stress. The material for transcriptome analysis was collected from different cucumber fruits at three key flowering stages. These samples were collected 5 days before flower opening (5 DBF), on the day of flower opening (0 DBF), and 5 days after flower opening (5 DAF). Each developmental stage was conducted with three biological replicates, which means that the samples originated from three different plants. Fruit peel was used to extract total RNA to construct the cDNA libraries. Clean data of 258.61 Gb were obtained from 36 samples through transcriptome sequencing. Clean data of at least 6.06 Gb were produced from each sample, with at least 92.92% of the data achieving a Q30 quality score. Next, mapping ratios ranging from 92.16 to 97.86% of the cucumber reference genome were aligned with the cleaned reads. Thus, this high mapping efficiency confirmed the suitability of the reference genome for subsequent bioinformatics analyses, including differential expression analysis (Table 1 and File S1).

### 2.6. Differentially Expressed Gene (DEG) Analysis

**Figure 4:**
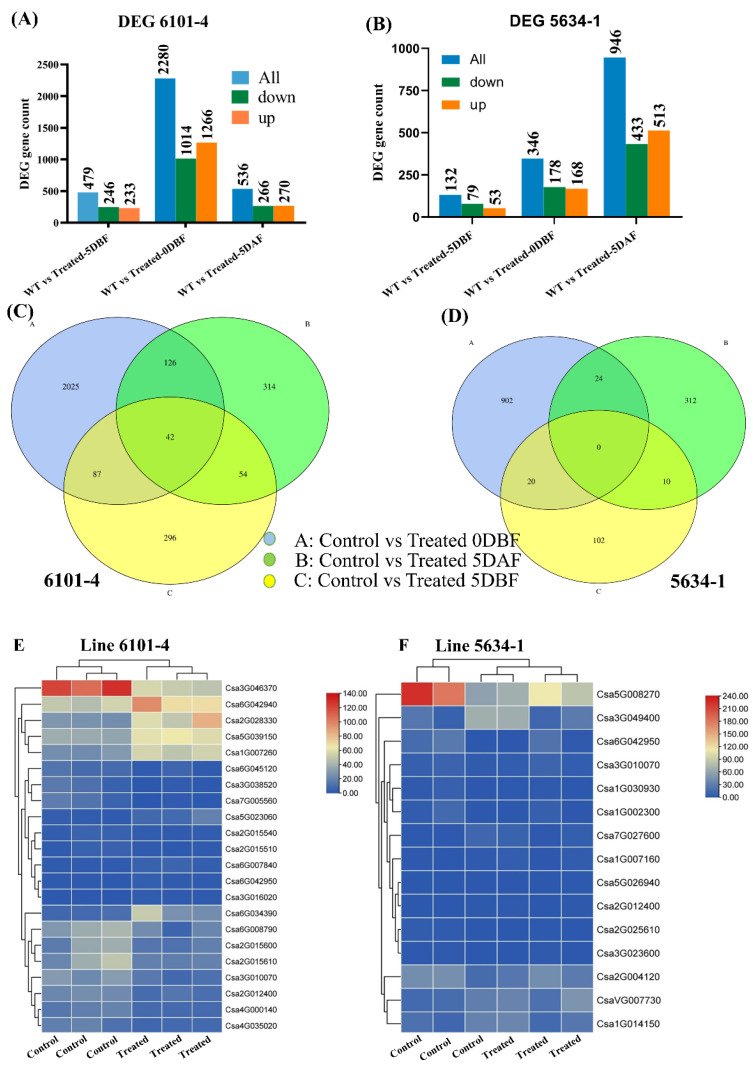

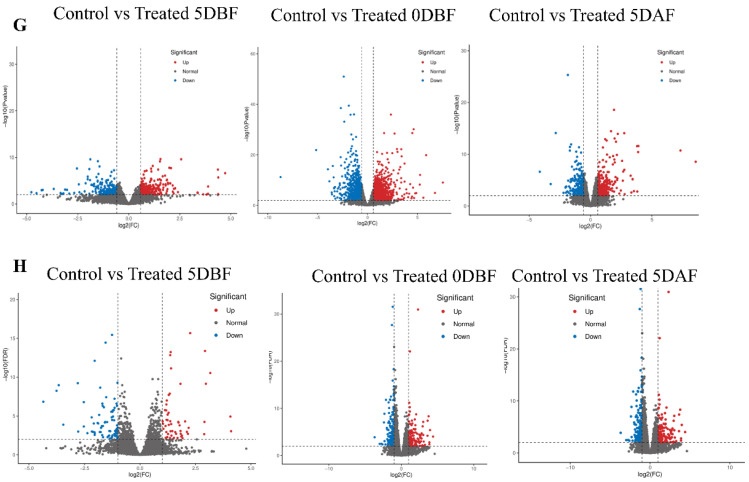
DEG analysis of 6101-4 and 5634-1 under control and treatment conditions. DEG counts of upregulated, downregulated, and total genes at 5 DBF, 0 DBF, and 5 DAF for (**A,B**). (**C,D**) Venn diagrams of DEG overlap between time points. Heatmap representation of differentially expressed genes in line 6101-4 and line 5634-1 (**E,F**). Volcano plots of 6101-4 (**G**) and (**H**) 5634-1 showing significant DEGs at 5 DBF, 0 DBF, and 5 DAF. Upregulated genes are indicated by red dots while downregulated genes are indicated by blue dots.

In the current study, transcriptome dynamics were analyzed, and candidate genes related to the trichome development mechanism in *C. sativus* were identified. Three different comparisons were generated from different samples, that is, the fruit peel of cucumber plants (control and treated) in both the 6101-4 and 5634-1 lines. Pairwise comparison analysis was performed, and the transcript levels of key unigenes among different samples were compared. In the comparison of control_5 DBF vs. treated_5 DBF, 479 unigenes were identified (233 upregulated and 246 downregulated); control_0 DBF vs. treated_0 DBF identified 2280 unigenes (1266 upregulated and 1014 downregulated); and control_5 DAF vs. treated_5 DAF identified 536 unigenes (270 upregulated and 266 downregulated) (Figure 4A) in line 6101-4. On the other hand, in line 5634-1, we also made three comparisons: control_5 DBF vs. treated_5 DBF identified 132 unigenes (53 upregulated and 79 downregulated), control_0 DBF vs. treated_0 DBF identified 346 unigenes (168 upregulated and 178 downregulated), and control_5 DAF vs. treated_5 DAF identified 946 unigenes (513 upregulated and 433 downregulated) (Figure 4B Table 2). The overlap between various samples was analyzed and presented using a Venn diagram, highlighting both unique and shared genes across the samples (Figure 4C,D). Additionally, a volcano plot was used to illustrate the distribution of differentially expressed genes between the control and treated plants at all three stages in both 6101-4 and 5634-1 (Figure 4G,H). The expression patterns of all the genes involved in these comparisons are detailed in Table 2 and Table S2.

### 2.7. GO and KEGG Enrichment Analysis

**Figure 5:**
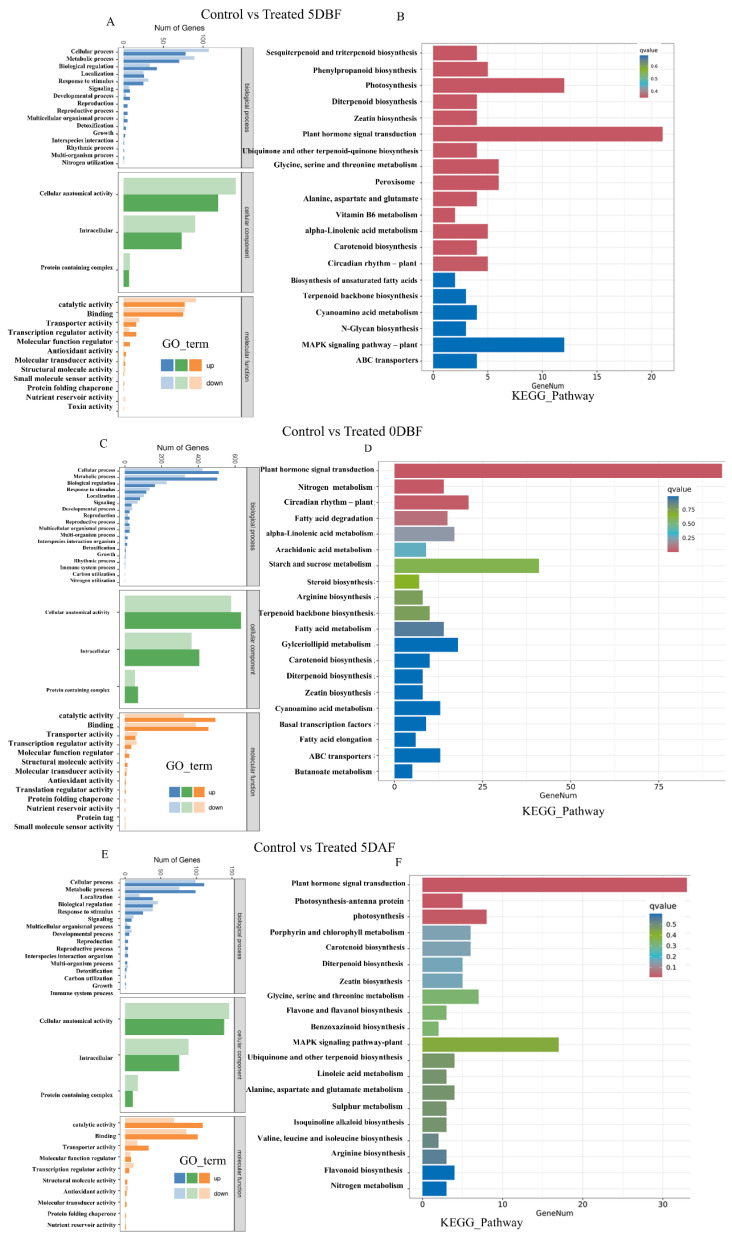
Gene Ontology (GO) analysis was conducted to compare control and treated cucumber plants at three fruit stages: 5 days before flowering (5 DBF) (**A**), at flowering (0 DBF) (**C**), and 5 days after flowering (5 DAF) (**E**). KEGG involved 5 days before flowering (5 DBF) (**B**), at flowering (0 DBF) (**D**), and 5 days after flowering (5 DAF) (**F**). The analysis highlighted the abundance of differentially expressed, enriched GO terms. The most enriched terms were categorized under the three main GO domains: biological process, cellular component, and molecular function.

Gene Ontology (GO) analysis was performed to categorize biological functions into three main subclasses: molecular function, biological process, and cellular component. The comparison of control vs. treated 5 DBF, control vs. treated 0 DBF, and control vs. treated 5 DAF represents 1797, 8847, and 2046 genes in 6101-4 and 480, 357, and 3987 genes in 5634-1, respectively. GO terms enriched with DEGs included cellular processes, metabolic processes, and biological regulation (Figure 5A,C,E). The most enriched terms for cellular components were cellular anatomical entity, intracellular components, and protein-containing complexes. In the case of molecular functions, GO terms enriched with DEGs included catalytic and binding activities (Figure 5A,C,E). GO term enrichment involved biosynthetic processes, defense mechanism regulation, cellular processes, cell wall, response to chemicals, signal transduction, cell communication, and regulation of many hormones in both inbred lines; these were highly enriched, and a heatmap showed that these genes have high, upregulated expression, and that this was greatly reduced/downregulated after the foliar application of chemicals at all three stages under investigation (Figure 4E,F). Using the KEGG pathway analysis of DEGs, at the 5 DBF stage, plant hormone signal transduction involving auxin and gibberellin was enriched, suggesting the initiation of developmental processes required for trichome initiation (Figure 5B). Moving to 0 DBF, the genes regulating ethylene and jasmonate pathways were found to be significantly upregulated, indicating their importance in the process of floral opening and stress response (Figure 5D). Ethylene and auxin signaling pathways were also enriched at 5 DAF, which is consistent with the previous observation and indicates their role in controlling trichome activity during fruit development (Figure 5F). Also, at the 5 DAF stage, secondary metabolism was enriched, including glycine, serine, and threonine metabolism, flavone and flavanol biosynthesis, and MAPK signaling pathway and flavonoid biosynthesis, which suggests that trichomes are engaged in the biosynthesis of bioactive compounds during fruit ripening (Figure 5F). Moreover, stress-responsive pathways, especially those linked to oxidative stress and the MAPK signaling pathway, were overrepresented at both 0 D and 5 DAF, suggesting that trichomes are beneficial during periods of stress. The AgNO_3_ and Na_2_S_2_O_3_ treatments significantly affected the main pathways, with AgNO_3_ stimulating ethylene signaling at the 0 DBF and 5 DAF stages and, consequently, the trichome activity and Na_2_S_2_O_3_ affecting the MAPK signaling pathway at 5 DAF, which points to the protective function of the trichomes. Furthermore, KEGG pathway enrichment analysis revealed 299 significantly enriched pathways overall (File S3).

### 2.8. Gene Expression Analysis by RT-PCR

**Figure 6:**
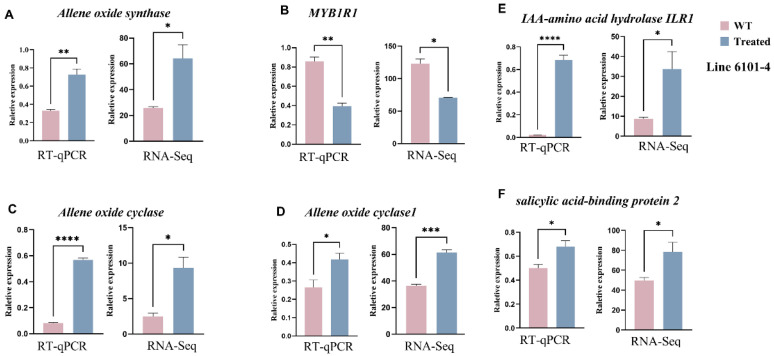
Validation of RNA-seq results using RT-qPCR for selected genes in line 6101-4. The left panels show the relative expression as determined by RT-qPCR, and the right panels present the RNA-seq data for comparison. (**A**–**F**) different genes from RNA-seq data of 6101-4. Error bars represent values of *n* = 3 samples. * *p* ≤ 0.05, ** *p* ≤ 0.01, *** *p* ≤ 0.001, **** *p* ≤ 0.0001.

**Figure 7:**
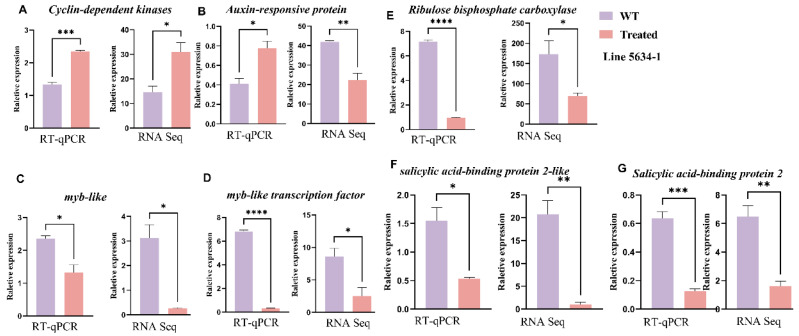
Validation of RNA-seq results using RT-qPCR for selected genes in line 5634-1. The results are displayed as mean ± SEM from three independent experiments. The left panels show the relative expression as determined by RT-qPCR, and the right panels present the RNA-seq data for comparison. (**A**–**G**) different genes from RNA-seq data of 5634-1. Error bars represent values of *n* = 3 samples. * *p* ≤ 0.05, ** *p* ≤ 0.01, *** *p* ≤ 0.001, **** *p* ≤ 0.0001.

The relative expression levels of various genes are shown in graphs of treatment vs. control under different conditions in two genetic lines of *C. sativus* L., 6101-4 and 5634-1. Two such treatments are silver nitrate (AgNO_3_) and sodium thiosulfate (STS). Gene expression was investigated to provide insight into how these substances influence gene regulation related to trichome formation and other physiological processes. We validated the differential expression of genes identified by RNA-seq analysis using independent RT-PCR samples at the same developmental stage as the samples used in the RNA-seq experiments. The RT-PCR results agreed with the DGE data in terms of reliability. In line 6101-4, genes such as *allene oxide synthase*, *ribosomal protein s7*, and *IAA-amino acid hydrolase ILR1* show significant upregulation, confirming their sensitivity to the applied conditions. In contrast, line 5634-1 exhibits notable downregulation of genes like *Arogenate dehydratase*, *myb-like* transcription, and *Cyclin*. The high statistical significance across most genes reinforces the reliability of the RNA-seq data, with RT-PCR results often showing more pronounced fold changes due to their sensitivity. We found a consistent trend across all genes, with the treated sample expressing significantly higher expression than the control sample. Both RT-PCR and RNA-seq datasets provided this evidence. Asterisks indicate the statistical significance of these differences; validation through RT-PCR confirmed the reliability of our RNA-seq data. The level of consistency between the two methods showed that the changes in gene expression that were observed in RNA-seq were real and the basis for further biological interpretation and analysis. Moreover, although these gene expression changes were statistically significant, they were reproducible across multiple experimental platforms (Figure 6 and Figure 7).

## 3. Discussion

Trichomes are essential for plant defense as they act as a physical barrier against biotic threats. Plants with higher trichome density exhibit enhanced resistance to herbivore feeding and reproduction, supported by the production of specialized secondary metabolites [28]. In addition to this physical change, trichome production of specialized metabolites as secondary chemicals provides a second line of defense [10,28,29]. As toxic and sticky constituents, nonvolatile metabolites may have gustative repellency [10], while volatile metabolites can act as olfactive deterrent. Moreover, certain molecules have antifungal and antibacterial features [30,31]. Categorized by the density and biosynthesis of specialized trichome metabolites, plants can help regulate both types of trichomes when exposed to biotic and abiotic stresses [32]. Studying trichome morphology and gene expression in *C. sativus* after treatment with AgNO_3_ and Na_2_S_2_O_3_ provides new insights into the molecular control of trichome initiation and development.

This study demonstrated that the foliar application of silver nitrate (AgNO_3_) and sodium thiosulfate (Na_2_S_2_O_3_) significantly enhanced trichome density and morphology in *C. sativus*. The finding of increased trichome density after chemical treatment is consistent with the well-established functions of trichomes in defense reactions and environmental adaptability [33]. Over all, our morphological data from both 6101-4 and 5643-1 also indicated that the fruit trichome density, stalk length, and base measurements were significantly enhanced [34]. Environmental variables and hormonal cues have a major impact on trichome growth in *C. sativus* and *Arabidopsis* [35]. Figure 1, Figure 2 and Figure 3 illustrate how chemical treatments increased trichome density and altered trichome stalk and base development. The results indicate that AgNO_3_ and Na_2_S_2_O_3_ play a role in initiating and promoting trichome growth by influencing hormone signal transduction pathways, including auxin and gibberellin, consistent with findings in other species [36].

RNA-seq analysis revealed that trichome development is governed by complex genetic networks involving transcription factors such as *GLABRA1* (*GL1*) and *TRANSPARENT TESTA GLABRA1* (*TTG1*) that have been shown to regulate trichome formation in previous studies of other crops, notably *A. thaliana*. Further, it has also been shown that the positive regulators *GLABRA1* (*GL1*), *GLABRA3* (*GL3*), *ENHANCER OF GLABRA3* (*EGL3*), and *TRANSPARENT TESTA GLABRA1* (*TTG1*) form an *MYB-bHLH-WDR* (*MBW*) activator complex to activate the expression of *GL2/TTG2* and *RBR/SIM*, thereby promoting trichome initiation [37,38,39,40].

The data showed upregulation of genes involved in secondary metabolism (e.g., flavonoid biosynthesis) and signaling pathways (e.g., MAPK signaling), suggesting a role for trichomes in bioactive compound biosynthesis during fruit ripening. These findings align with earlier studies, indicating that chemical treatments significantly affect gene expression related to trichome initiation [41,42]. The initial development of trichomes is one of the most important developmental stages, and its modulation is dependent on ethylene. The results of this study showed that silver ion treatment increased trichome density and changed trichome shape, consistent with the notion that silver ions disrupt ethylene function [43]. In a previous investigation of *G. hirsutum*, it was discovered that silver ions can change the structure of fibers to be similar to trichomes [44]. This could lead to changes in the gene cycle of expression associated with trichome production. Differential gene expression in response to pharmacological treatments such as *MYB1R1* and *allene oxide synthase* (*AOS*) emphasize the intricate regulatory networks governing trichome initiation. These genes could be linked to plant defense mechanisms and trichome development in response to foliar chemical treatments. In cultivar 5634-1, genes including *ribulose bisphosphate carboxylase/oxygenase activase* and *cyclin-dependent kinase regulatory subunit* were greatly enhanced, suggesting that these genes may play important roles in cell cycle control and photosynthesis following chemical treatment. Strong ethylene-acting inhibitors, such as silver nitrate, control plant hormone pathways related to development and sex expression [45].

The hormonal signaling pathways modulated by chemical treatments align with findings in other plant species. For example, it has been shown that the *MYC2* transcription factor, a part of the jasmonate signaling pathway in *S. lycopersicum*, regulates trichome and secondary metabolite production [46,47]. *MYB*-related genes were increased in *C. sativus* when they were treated with Na_2_S_2_O_3_ and AgNO_3_, suggesting that these chemicals may activate similar signaling pathways and cause trichome formation. Sodium thiosulfate is used as a sulfur donor, has not been well studied during trichome growth, and can affect many metabolic pathways [48]. These results indicate that sulfur application may be involved in the control of trichome development and the effects of Na_2_S_2_O_3_ on trichome number and phenotype. Studies on the growth of cucumber trichomes in A. thaliana have also shown that the growth of cucumber trichomes is affected by sulfur deficiency; thus, sulfur is required for the synthesis of chemicals involved in trichome initiation [42]. For instance, studies on tomato glandular trichomes [49] demonstrated that the administration of jasmonic acid (JA) changed the patterns of gene expression and increased trichome density, as did AgNO_3_ and Na_2_S_2_O_3_ in *C. sativus.* This suggests that identical hormonal and signaling pathways may control trichome production in a wide variety of plant species. This study identified regulatory genes, which, together with the molecular pathways discussed in this work, contribute to the understanding of the molecular pathways associated with ethylene signaling. In a study of gene expression patterns, a relationship between these morphological changes and the upregulation of genes involved in the regulation of the cell cycle, auxin-responsive genes, and jasmonic acid synthesis, as well as the downregulation of specific transcription factors, was observed. Furthermore, these results provide new insights into the molecular mechanisms involved in trichome initiation and development in *C. sativus*.

The enhancement of trichome density and gene expression regulation by chemical treatments highlights their potential for improving cucumber breeding and resilience against environmental stresses. The identified regulatory genes and pathways provide new insights into the molecular control of trichome development in *C. sativus*. Future studies could explore the long-term impacts of these chemical treatments on plant physiology and the role of sulfur in trichome-associated metabolic pathways. This study demonstrates the significant influence of AgNO_3_ and Na_2_S_2_O_3_ on trichome morphology and gene expression in *C. sativus*. By elucidating the molecular mechanisms underlying trichome development, it contributes to our understanding of plant defense and environmental adaptability. The findings also highlight the potential of chemical treatments in agricultural practices, paving the way for enhanced crop protection and productivity.

## 4. Materials and Methods

### 4.1. Selection and Cultivation of Two Inbred Lines

Specific traits or characteristics of interest were selected from two inbred cucumber lines, i.e., 6101-4 and 5634-1. To achieve controlled and optimal growth, the lines were grown in a greenhouse in February and March. To achieve plant health and uniformity, all plants were grown in a greenhouse under natural sunlight in the experimental field of the China Agricultural University in Beijing.

### 4.2. Phenotypic and Scanning Electron Microscopy Data Collection

Scanning electron microscopy (SEM) was performed on two inbred cucumber lines, 5634-1 and 6101-4, including both treated and control plants, at three fruit developmental stages: 5 days before flowering (5 DBF), the day of flowering (0 DBF), and 5 days after flowering (5 DAF). Samples were fixed with 2.5% glutaraldehyde at 4 °C for 24 h, washed with PBS (pH 7.2), and post-fixed with 1% OsO4. High-resolution images of trichomes were obtained from fruits using a Hitachi S-4700 SEM (Hitachi, Ltd. 6-6, Marunouchi 1-chome, Chiyoda-ku, Tokyo, Japan) with a 2 kV accelerating voltage [6]. Trichome density, length, and width were measured across the three developmental stages in both treated and control plants. Comparisons of trichome morphology, including density, size, shape, and surface features, were made between treated and untreated samples, highlighting differences under different treatment conditions.

### 4.3. Foliar Application of Sodium Thiosulphate (Na_2_S_2_O_3_) and Silver Nitrate (AgNO_3_)

The combined solutions of silver nitrate (AgNO_3_) and sodium thiosulfate (Na_2_S_2_O_3_) were prepared, and both 0.2 g of AgNO_3_ and 1.6 g of Na_2_S_2_O_3_ were dissolved in 1 L of distilled water. In the control, there were no treatments. The solutions were stored in the dark until further use. These solutions were applied to the top of the plant as a foliar application. When 2 to 3 true leaves emerged, treatments were applied. The first female fruit was selected for transcriptome analysis and phenotypic observation. While the treatment was applied at approximately 200 mL to 300 mL per 10 plants, we ensured consistent application by spraying it uniformly, fully covering the entire foliage evenly. The application was repeated three times to ensure consistent exposure. These chemicals mainly induce male flowers and significantly affect trichomes on cucumber fruits, leading to an increase in trichome density in treated plants compared to that in the control.

### 4.4. Sample Collection for Sequencing

We collected cucumber fruit peels at three developmental stages—5 days before flower opening (5 DBF), 0 days before flower opening (0 DBF), and 5 days after flower opening (5 DAF—from both control and treated plants. The plant total RNA was extracted using the RNAprep Pure Plant Kit (Tiangen, Beijing, China) according the instructions provided by the manufacturer. To ensure biological repeatability, the samples were sent for high-throughput Illumina sequencing on the Biomarker (Baimaike) BMK platform Beijing, China (project issue number: BMK230519-BJ971-ZX01-0103). Genetic variants, gene expression patterns, and possible regulatory elements were identified from the processed sequencing data, which were reflected in their function in trichome development.

### 4.5. RNA Extraction and Library Preparation

RNA was extracted from total RNA using the RNAprep Pure Plant Kit (Tiangen, Beijing, China), according to the manufacturer’s protocol. A NanoDrop 2000 spectrophotometer (Thermo Fisher Scientific, Wilmington, DE, USA) was used to measure RNA concentration and purity, and an Agilent Bioanalyzer 2100 (Agilent Technologies, Santa Clara, CA, USA) with an RNA Nano 6000 Assay Kit was used to assess RNA integrity. Isolation of mRNA from total RNA was performed using poly T oligo-go-attached magnetic beads. First-strand cDNA was synthesized with fragmented mRNA as template and random hexamers as primers, followed by second-strand synthesis with the addition of PCR buffer, dNTPs, RNase H, and DNA polymerase I. The PCR products were purified using the AMPure XP system and the quality of the libraries was assessed using an Agilent Bioanalyzer 2100.

### 4.6. Analysis of Transcriptome Sequencing

The datasets were then sequenced, and adaptor sequences and low-quality sequence reads were removed. After data processing, raw sequences were transformed into clean reads. Using HISAT (v 2.1.0), (http://ccb.jhu.edu/software/hisat2/index.shtml, accessed on 1 November 2024) these clean reads were mapped to the reference genome sequence of Cu-cumis_sativus. ChineseLong_v3.genome.fa [50]. Q20, Q30, GC content, and the sequence duplication levels of the clean data were also calculated. High-quality clean data were used for all the downstream analyses. Fragments per kilobase of transcript per million fragments mapped (FPKM) was used to quantify gene expression levels. Differential expression analysis of the two conditions/groups was performed using DESeq2 [51]. Statistical routines for determining differential expression in digital gene expression data are provided in DESeq2 (https://bioconductor.org/packages/release/bioc/html/DESeq2.html, accessed on 1 November 2024) based on a negative binomial model. Benjamini and Hochberg’s approach was used to adjust the resulting *p*-values to control the false discovery rate. Differentially expressed genes were assigned by DESeq2 when genes had an adjusted *p* value < 0.01. Heatmaps were constructed using TBtools II v2.118 software [52] for gene visualization.

### 4.7. Gene Ontology and KEGG Pathway Analysis

GO enrichment analysis was conducted to categorize the genes into three main categories: biological processes, cellular components, and molecular functions. Using the GO seq R package 10 July 2023 (http://www.rproject.org, accessed on 1 November 2024), we analyzed differentially expressed genes (DEGs) using Wallenius non-central hypergeometric distribution to account for gene length bias [53]. A KEGG pathway analysis was performed. The Kyoto Encyclopedia of Genes and Genomes (KEGG) is a comprehensive database for high-level biological functions and system utility at cellular and organismal levels, from molecular data, especially from large-scale data such as genome sequencing. The statistical enrichment of DEGs in KEGG pathways was analyzed using KOBAS software v3.0.3 [54].

### 4.8. Quantitative Real-Time PCR (qRT-PCR) for Data Validation

Specific genes of interest identified from sequencing data were selected for further validation. The plant total RNA was extracted using the RNAprep Pure Plant Kit (Tiangen, Beijing, China) according the instructions provided by the manufacturer, and reverse transcription was performed using the PrimeScript reagent kit and gDNA Eraser (TaKaRa, Shiga, Japan), following the manufacturer’s protocol. RT-qPCR was conducted on an ABI 7500 Real-Time PCR System (Applied Biosystems, Waltham, MA, USA) in 380-well plates using SYBR Premix Ex Taq (TaKaRa). Three biological replicates and three technical replicates were included for each cDNA sample and primer–pair combination. Ubiquitin was used as the internal control gene [55]. RT-qPCR was conducted to quantify the expression levels of the target genes by comparing the treated and untreated samples and two inbred lines. The results were analyzed to determine the fold changes in gene expression, with fluorescence curves indicating positive or negative regulation. The gene-specific RT-qPCR primers are listed in (File S4).

### 4.9. Data Analysis and Interpretation

Phenotypic, SEM, and gene expression data were integrated to provide a comprehensive understanding of how sodium thiosulfate and silver nitrate treatments affect trichome density and morphology. For all expression levels and phenotypic analyses, statistical significance was determined using Statistix 8.1 using two-factor LSD significant difference test and GraphPad prism 9. The combined data help elucidate the underlying genetic and molecular mechanisms that regulate trichome development in response to chemical treatments.

## 5. Conclusions

This work shows that male flower inducers have a marked impact on trichome development in two inbred lines of *C. sativus* L., with clear changes in the trichome sharpness, structure, density, and number. We therefore uncovered the key DEGs related to hormone regulation, intracellular transport, and defensive response to trichome development through RNA sequencing. The identification of specific genes including *allene oxide synthase* and *MYB1R1* transcription factors reveals specific molecular targets that can be manipulated to improve trichome properties. The findings of this study are, however, most significant for agricultural uses. For example, the application of silver nitrate (AgNO_3_) and sodium thiosulfate (Na_2_S_2_O_3_) to foliage may be fine-tuned to enhance the resistance of cucumber to pests and other stress factors. Further studies should be directed at furthering the understanding of how best to apply the trichome-related compounds in terms of concentration and duration of exposure. Moreover, the application of CRISPR/Cas9 and other gene editing tools to manipulate the above-mentioned regulatory genes for defense mechanisms and adaptability could make it possible to develop new improved cultivars of cucumber.

Future work should also examine the effects of increased trichome density on plant health, yield, and quality in the future. The relationship between the trichome-associated genes and environmental factors, for instance, temperature, humidity, and the type of soil, could be further explored to understand how trichome development could be helpful in various agricultural conditions. Further research on the efficacy of the secondary metabolites derived from trichomes in the defense against pests and pathogens will enhance this work, with possible biopesticide prospects. The biochemical pathways that are affected by trichome development and secondary metabolite biosynthesis offer potential for commercial applications such as pest control crops and increased crop yield. This study therefore forms a basis for breeding strategies for enhanced trichome characteristics in cucumbers in a bid to achieve better trichome possibilities in sustainable farming. Further studies should be directed towards the use of interdisciplinary research that combines molecular biology, agronomy, and environmental sciences to better understand the potential of trichome biology in cucumbers and other crops of economic importance. It could also play a big role in the fight for food security and sustainability all over the world.

## Figures and Tables

**Table 1:** An overview of sequencing assembly in fruit peel of cucumber fruit.

Sample	Total Clean Reads (M)	Clean Reads Q20 (%)	Clean Reads Q30 (%)	Total Mapping (%)	Unique Mapping (%)
Treated-5 DBF-6101-4	59.65	97.96	94.11	95.97	91.99
Treated-0 DBF-6101-4	43.45	97.53	93.05	97.16	94.06
Treated-5 DAF-6101-4	57.38	97.81	93.84	96.93	94.02
Contro-5 DBF-6101-4	49.10	97.73	93.52	97.61	94.65
Contro-0 DBF-6101-4	42.31	97.73	93.51	96.44	92.72
Contro-5 DAF-6101-4	43.96	97.73	93.51	97.46	94.60
Treated-5 DBF-5634-1	45.01	97.59	93.21	97.07	93.65
Treated-0 DBF-5634-1	42.88	97.71	93.48	97.30	94.25
Treated-5 DAF-5634-1	47.51	97.76	93.58	97.12	94.57
Contro-5 DBF-5634-1	43.72	97.61	93.31	97.16	85.61
Contro-0 DBF-5634-1	46.22	98.04	94.24	97.35	94.42
Contro-5 DAF-5634-1	41.76	97.77	93.66	96.96	93.37

**Table 2:** Differentially expressed genes (DEGs) in cucumber lines across flowering stages and treatments.

Cucumber Lines	Comparison	Total Unigenes Identified	Upregulated	Downregulated
6101-4	control_5 DBF vs. treated_5 DBF	479	233	246
6101-4	control_0 DBF vs. treated_0 DBF	2280	1266	1014
6101-4	control_5 DAF vs. treated_5 DAF	536	270	266
5634-1	control_5 DBF vs. treated_5 DBF	132	53	79
5634-1	control_0 DBF vs. treated_0 DBF	346	168	178
5634-1	control_5 DAF vs. treated_5 DAF	946	513	433

## Data Availability

The data presented in this study are available in the Sequence Read Archive (SRA) database in NCBI (Accession No. PRJNA1185714).

## Supplementary Materials

The following supporting information can be downloaded at: https://www.mdpi.com/article/10.3390/ijms26031298/s1.

## References

[1] Renner S.S., Schaefer H., Grumet R., Katzir N., Garcia-Mas J. (2016). Genetics and Genomics of Cucurbitaceae.

[2] Chomicki G., Schaefer H., Renner S.S. (2020). Origin and Domestication of Cucurbitaceae Crops: Insights from Phylogenies, Genomics and Archaeology. New Phytol..

[3] Qing Z., Shi Y., Han L., Li P., Zha Z., Liu C., Liu X., Huang P., Liu Y., Tang Q. (2022). Identification of Seven Undescribed Cucurbitacins in *Cucumis sativus* (Cucumber) and Their Cytotoxic Activity. Phytochemistry.

[4] Xu Y., Zhang H., Zhong Y., Jiang N., Zhong X., Zhang Q., Chai S., Li H., Zhang Z. (2022). Comparative Genomics Analysis of BHLH Genes in Cucurbits Identifies a Novel Gene Regulating Cucurbitacin Biosynthesis. Hortic. Res..

[5] Liu X., Wang L., Liu L., Guo Y., Ren H. (2011). Alleviating Effect of Exogenous Nitric Oxide in Cucumber Seedling against Chilling Stress. African J. Biotechnol..

[6] Chen C., Liu M., Jiang L., Liu X., Zhao J., Yan S., Yang S., Ren H., Liu R., Zhang X. (2014). Transcriptome Profiling Reveals Roles of Meristem Regulators and Polarity Genes during Fruit Trichome Development in Cucumber (*Cucumis Sativus* L.). J. Exp. Bot..

[7] Yamamoto Y., Hayashi M., Kanamaru T., Watanabe T., Mametsuka S., Tanaka Y. (1989). Studies on Bloom on the Surface of Cucumber [*Cucumis sativus*] Fruits, 2: Relation between the Degree of Bloom Occurrence and Contents of Mineral Elements. Bulletin of the Fukuoka Agricultural Research Center. Series B Horticulture.

[8] Li Y., Wen C., Weng Y. (2013). Fine Mapping of the Pleiotropic Locus B for Black Spine and Orange Mature Fruit Color in Cucumber Identifies a 50 Kb Region Containing a R2R3-MYB Transcription Factor. Theor. Appl. Genet..

[9] Wagner G.J. (1991). Secreting Glandular Trichomes: More than Just Hairs. Plant Physiol..

[10] Werker E. (2000). Trichome Diversity and Development. Advances in Botanical Research. Plant Trichomes.

[11] Eisner T., Eisner M., Hoebeke E.R. (1998). When Defense Backfires: Detrimental Effect of a Plant’s Protective Trichomes on an Insect Beneficial to the Plant. Proc. Natl. Acad. Sci. USA.

[12] Riddick E.W., Simmons A.M. (2014). Do Plant Trichomes Cause More Harm than Good to Predatory Insects?. Pest Manag. Sci..

[13] Glas J.J., Schimmel B.C.J., Alba J.M., Escobar-Bravo R., Schuurink R.C., Kant M.R. (2012). Plant Glandular Trichomes as Targets for Breeding or Engineering of Resistance to Herbivores. Int. J. Mol. Sci..

[14] Lange B.M., Ahkami A. (2013). Metabolic Engineering of Plant Monoterpenes, Sesquiterpenes and Diterpenes—Current Status and Future Opportunities. Plant Biotechnol. J..

[15] Loughner R., Goldman K., Loeb G., Nyrop J. (2008). Influence of Leaf Trichomes on Predatory Mite (*Typhlodromus pyri*) Abundance in Grape Varieties. Exp. Appl. Acarol..

[16] Vosman B., Kashaninia A., van’t Westende W., Meijer-Dekens F., van Eekelen H., Visser R.G.F., de Vos R.C.H., Voorrips R.E. (2019). QTL Mapping of Insect Resistance Components of Solanum Galapagense. Theor. Appl. Genet..

[17] Shi P., Fu X., Shen Q., Liu M., Pan Q., Tang Y., Jiang W., Lv Z., Yan T., Ma Y. (2018). The Roles of Aa MIXTA 1 in Regulating the Initiation of Glandular Trichomes and Cuticle Biosynthesis in Artemisia Annua. New Phytol..

[18] Tissier A. (2012). Glandular Trichomes: What Comes after Expressed Sequence Tags?. Plant J..

[19] Arpan H. (1974). The Researches on Effect of 2-Chloroethylophosphonic Acid (Ethrel) on Gender Appearance and Some Other Characters of Cucumber. Fac. Agric. Publ..

[20] Dai D., Wang L., Liu Y., Chu M., Wang J., Ji P., Sheng Y. (2022). Screening of Key Genes Promoting Stamen Formation Induced by Silver Nitrate in Gynoecious Melon. SSRN.

[21] Hu W., Su Y., Zhou J., Zhu H., Guo J., Huo H., Gong H. (2022). Foliar Application of Silicon and Selenium Improves the Growth, Yield and Quality Characteristics of Cucumber in Field Conditions. Sci. Hortic..

[22] Owen L.C. (2023). Pollen Quality, Storage, Breeding, and Cytology of Cannabis sativa L..

[23] Chandler J.W. (2011). The Hormonal Regulation of Flower Development. J. Plant Growth Regul..

[24] Beyer E.M. (1976). A Potent Inhibitor of Ethylene Action in Plants. Plant Physiol..

[25] Rodrıguez F.I., Esch J.J., Hall A.E., Binder B.M., Schaller G.E., Bleecker A.B. (1999). A Copper Cofactor for the Ethylene Receptor ETR1 from *Arabidopsis*. Science.

[26] Zhao X.-C., Qu X., Mathews D.E., Schaller G.E. (2002). Effect of Ethylene Pathway Mutations upon Expression of the Ethylene Receptor ETR1 from Arabidopsis. Plant Physiol..

[27] Binder B.M., Rodriguez F.I., Bleecker A.B., Patterson S.E. (2007). The Effects of Group 11 Transition Metals, Including Gold, on Ethylene Binding to the ETR1 Receptor and Growth of Arabidopsis Thaliana. FEBS Lett..

[28] Kahana A., Silberstein L., Kessler N., Goldstein R.S., Perl-Treves R. (1999). Expression of ACC Oxidase Genes Differs among Sex Genotypes and Sex Phases in Cucumber. Plant Mol. Biol..

[29] Duan Q.-H., Wang D.-H., Xu Z.-H., Bai S.-N. (2008). Stamen Development in Arabidopsis Is Arrested by Organ-Specific Overexpression of a Cucumber Ethylene Synthesis Gene CsACO_2_. Planta.

[30] Singh G.P., Singh R.K. (1984). Chemical Sex Modification and Its Effect on Fruiting in Cucumber (*Cucumis sativus* L.). South Indian Hortic..

[31] Kaur H., Manchanda P., Dhall R.K. (2024). Effect of Foliar Application of Plant Growth Regulators on Expression of Flowering Genes in Cucumber (*Cucumis sativus* L.). Nucleus.

[32] Karakaya D., PADEM H. (2011). The Effects of Silver Nitrate Applications on the Flower Quantity of Cucumbers (*Cucumis sativus* L.). Not. Bot. Horti Agrobot. Cluj-Napoca.

[33] Levin D.A. (1973). The Role of Trichomes in Plant Defense. Q. Rev. Biol..

[34] Johnson H.B. (1975). Plant Pubescence: An Ecological Perspective. Bot. Rev..

[35] Crouzet J., Roland J., Peeters E., Trombik T., Ducos E., Nader J., Boutry M. (2013). NtPDR1, a Plasma Membrane ABC Transporter from Nicotiana Tabacum, Is Involved in Diterpene Transport. Plant Mol. Biol..

[36] Yang J., Li Y.-C., Zhou X.-R., Xu X.-J., Fu Q.-Y., Liu C.-Z. (2018). Two Thymol Derivatives from the Flower Buds of Lonicera Japonica and Their Antibacterial Activity. Nat. Prod. Res..

[37] Paudel S., Lin P.-A., Foolad M.R., Ali J.G., Rajotte E.G., Felton G.W. (2019). Induced Plant Defenses against Herbivory in Cultivated and Wild Tomato. J. Chem. Ecol..

[38] Kaur I., Kariyat R. (2023). Trichomes Mediate Plant–Herbivore Interactions in Two Cucurbitaceae Species through Pre-and Post-Ingestive Ways. J. Pest Sci..

[39] Watts S., Kariyat R. (2021). Morphological Characterization of Trichomes Shows Enormous Variation in Shape, Density and Dimensions across the Leaves of 14 Solanum Species. AoB Plants.

[40] Xue S., Dong M., Liu X., Xu S., Pang J., Zhang W., Weng Y., Ren H. (2019). Classification of Fruit Trichomes in Cucumber and Effects of Plant Hormones on Type II Fruit Trichome Development. Planta.

[41] Lv D., Wang G., Zhang Q., Yu Y., Qin P.-C., Pang J.-A., Sun J.-X., Zhang K.-Y., He H.-L., Cai R. (2022). Comparative Transcriptome Analysis of Hard and Tender Fruit Spines of Cucumber to Identify Genes Involved in the Morphological Development of Fruit Spines. Front. Plant Sci..

[42] Zhang L., Lv D., Pan J., Zhang K., Wen H., Chen Y., Du H., He H., Cai R., Pan J. (2021). A SNP of HD-ZIP I Transcription Factor Leads to Distortion of Trichome Morphology in Cucumber (*Cucumis sativus* L.). BMC Plant Biol..

[43] Kundan M., Gani U., Nautiyal A.K., Misra P. (2019). Molecular Biology of Glandular Trichomes and Their Functions in Environmental Stresses. Molecular Approaches in Plant Biology and Environmental Challenges.

[44] Jain S., Nidhi N., Kale S., Rathod M., Dhurve L., Mehara H., Baidya B.K. (2023). A Comprehensive Review on Role of Bio-Regulators in the Growth and Development of Fruit and Vegetable Crops. Int. J. Environ. Clim. Change.

[45] Juturu V.N., Mekala G.K., Kirti P.B. (2015). Current Status of Tissue Culture and Genetic Transformation Research in Cotton (*Gossypium* Spp.). Plant Cell Tissue Organ Cult..

[46] Spyropoulou E.A., Haring M.A., Schuurink R.C. (2014). RNA Sequencing on Solanum Lycopersicum Trichomes Identifies Transcription Factors That Activate Terpene Synthase Promoters. BMC Genom..

[47] Yu L., Zhang Y., Ding Q., Wang H., Meng X., Fan H., Yu Y., Cui N. (2024). The SlMYC1-TOR Module Regulates Trichome Formation and Terpene Biosynthesis in Tomatoes (*Solanum lycopersicum* L.). J. Plant Growth Regul..

[48] Váňová L. (2009). Of Dissertation: Use of in vitro Culture for Risk Assessment of Pahs in Plants. J. Exp. Bot.

[49] Chandra-kuntal K. (2022). Ethylene and ROS Crosstalk in Plant Developmental Processes. Ethylene in Plant Biology.

[50] Goossens J., Mertens J., Goossens A. (2017). Role and Functioning of BHLH Transcription Factors in Jasmonate Signalling. J. Exp. Bot..

[51] Pertea M., Pertea G.M., Antonescu C.M., Chang T.-C., Mendell J.T., Salzberg S.L. (2015). StringTie Enables Improved Reconstruction of a Transcriptome from RNA-Seq Reads. Nat. Biotechnol..

[52] Love M.I., Huber W., Anders S. (2014). Moderated Estimation of Fold Change and Dispersion for RNA-Seq Data with DESeq2. Genome Biol..

[53] Young M.D., Wakefield M.J., Smyth G.K., Oshlack A. (2010). Gene Ontology Analysis for RNA-Seq: Accounting for Selection Bias. Genome Biol..

[54] Mao X., Cai T., Olyarchuk J.G., Wei L. (2005). Automated Genome Annotation and Pathway Identification Using the KEGG Orthology (KO) as a Controlled Vocabulary. Bioinformatics.

[55] Wan H., Zhao Z., Qian C., Sui Y., Malik A.A., Chen J. (2010). Selection of Appropriate Reference Genes for Gene Expression Studies by Quantitative Real-Time Polymerase Chain Reaction in Cucumber. Anal. Biochem..

